# Relating resting-state fMRI and EEG whole-brain connectomes across frequency bands

**DOI:** 10.3389/fnins.2014.00258

**Published:** 2014-08-28

**Authors:** Fani Deligianni, Maria Centeno, David W. Carmichael, Jonathan D. Clayden

**Affiliations:** Neuroimaging and Neural Networks, University College London Institute of Child HealthLondon, UK

**Keywords:** brain connectivity, simultaneous EEG-fMRI, resting-state brain connectomes, statistical prediction, band limited power

## Abstract

Whole brain functional connectomes hold promise for understanding human brain activity across a range of cognitive, developmental and pathological states. So called resting-state (rs) functional MRI studies have contributed to the brain being considered at a macroscopic scale as a set of interacting regions. Interactions are defined as correlation-based signal measurements driven by blood oxygenation level dependent (BOLD) contrast. Understanding the neurophysiological basis of these measurements is important in conveying useful information about brain function. Local coupling between BOLD fMRI and neurophysiological measurements is relatively well defined, with evidence that gamma (range) frequency EEG signals are the closest correlate of BOLD fMRI changes during cognitive processing. However, it is less clear how whole-brain network interactions relate during rest where lower frequency signals have been suggested to play a key role. Simultaneous EEG-fMRI offers the opportunity to observe brain network dynamics with high spatio-temporal resolution. We utilize these measurements to compare the connectomes derived from rs-fMRI and EEG band limited power (BLP). Merging this multi-modal information requires the development of an appropriate statistical framework. We relate the covariance matrices of the Hilbert envelope of the source localized EEG signal across bands to the covariance matrices derived from rs-fMRI with the means of statistical prediction based on sparse Canonical Correlation Analysis (sCCA). Subsequently, we identify the most prominent connections that contribute to this relationship. We compare whole-brain functional connectomes based on their geodesic distance to reliably estimate the performance of the prediction. The performance of predicting fMRI from EEG connectomes is considerably better than predicting EEG from fMRI across all bands, whereas the connectomes derived in low frequency EEG bands resemble best rs-fMRI connectivity.

## 1. Introduction

Large scale networks with correlated time courses have been consistently identified in the resting brain with functional Magnetic Resonance Imaging (fMRI) (Beckmann and Smith, 2004; Varoquaux et al., 2010b), and electroencephalography (EEG) (Tagliazucchi et al., 2012) and magnetoencephalography (MEG) (Brookes et al., 2011a,b). Spontaneous neural fluctuations exhibit consistent correlation structures over a wide range of spatial and temporal scales and they constitute a prominent energy-consuming feature of the brain (Schölvinck et al., 2013; Smith et al., 2013). Several studies highlight their significance in modulating brain function and task efficiency (Bonnelle et al., 2012). Furthermore, abnormalities of resting-state (rs) connectivity have been also implicated in several neurological diseases, including epilepsy, schizophrenia, attention deficit hyperactivity disorder, Alzheimers disease, stroke and traumatic brain injury (Zhang and Raichle, 2010).

Multi-modal approaches and in particular combined electrophysiological measures with fMRI offer the opportunity to observe neurophysiological events in high temporal and spatial resolution. fMRI data are acquired as series of volumetric images, typically obtained every few seconds, that represent blood oxygen level-dependent (BOLD) contrast. This mechanism is related to the delivery of blood to active neuronal tissue and hence it allows indirect inference on brain function. This places a limit on the temporal resolution of neuronal fluctuations observed with rs-fMRI and complicates the interpretation of the estimated connectivity. On the other hand, in EEG, multiple electrodes are placed on the scalp to measure spontaneous electrical activity. Although temporal resolution of EEG is on the scale of milliseconds, the localization of the signal involves sophisticated algorithms and a priori models for both the source and the volume conductor and yet it only achieves accuracy in the range of 1–2 cm (Kaiboriboon et al., 2012).

To fully exploit the advantages of combining multi-modal information, we need to understand the relationship between the underlying modalities as well as and their neurophysiological origins (Laufs et al., 2008). Pioneering intracranial recordings have established a link between the local BOLD signal and the underling neuronal activity (Logothetis et al., 2001; Logothetis, 2003; Mukamel et al., 2005; Magri et al., 2012; Chang et al., 2013). However, these studies do not capture the cooperative processes underpinning brain function that involves whole brain organization. Furthermore, they are invasive and their application is limited in animals and in specific patient cohorts with neurological abnormalities. We are interested in examining the relationship of brain connectomes derived from simultaneous recordings of fMRI and EEG in rest.

Specific EEG features from the scalp, such as occipital alpha and beta bands have been related to RSN observed with fMRI (Laufs et al., 2003; Moosmann et al., 2003). These studies have revealed networks with a large degree of commonality with resting state networks such as the default mode and attentional networks. Investigating neuronal activity in different frequency bands has attracted considerable attention because it is hypothesized to subserve different roles and originate from anatomically separated but functionally related brain regions. For instance, band-limited gamma effects have been linked to enhanced neural communication, while alpha oscillations have been related to functional inhibition (Scheeringa et al., 2011). These studies along with studies of seed-based analysis (de Pasquale et al., 2010; Brookes et al., 2011a) provided insight on the relationship of BOLD fMRI and EEG within specific networks. One major limitation of methodologies based on the topographic electrophysiological signatures of RSN is that the agreement between RSN observed with fMRI and EEG relies on the spatial relationship of the extracted networks (Razavi et al., 2013). This process depends on thresholding and it does not provide information about the intra-cerebral location of the EEG signal nor about the relationship between specific RSN connections and EEG rhythms (Jann et al., 2010).

Recently, Brookes et al. derived resting state networks in a range of band-limited power (BLP) frequency ranges using MEG and investigated their relationship with the rs-fMRI (Brookes et al., 2011a,b). They used a beamforming source localization to map the MEG signal from sensor space to source/voxel space. Source localization provides spatial information that allows one to draw direct regions' correspondence across subjects. Subsequently, temporal independent component analysis (ICA) of the Hilbert envelope of the MEG signal highlighted brain networks that closely resemble known rs-fMRI networks (Brookes et al., 2011b). This confirmed further the neurophysiological origin of the resting-state networks that emerge in fMRI data (Smith et al., 2009).

However, these comparisons were based on the spatial agreement between the temporal ICA components estimated across MEG frequency bands and the spatial ICA components derived from the analysis of rs-fMRI data (Brookes et al., 2011b). This approach is limited in that it uses non-simultaneous acquisition of MEG and fMRI data without any guarantee that differences in these environments (e.g., motion, auditory input) would not affect the outcome. Furthermore, temporal and spatial ICA can have diverging results, depending upon the spatiotemporal characteristics of the underlying sources. Whereas spatial agreement between the two maps is reassuring, further information about how the covariance structure between EEG and fMRI signals differ is needed to fully understand their relationship. In particular knowledge of the key connections that contribute to the prediction of one connectome from the other may give insight into the parts of the network that are frequency specific and common to each modality.

We develop a statistical framework to learn the relationship between connectomes derived from rs-fMRI and the BLP spectrums of simultaneous source-localized EEG recordings. To achieve this we relate the covariance structure of the Hilbert envelope of the source localized electrophysiological signal to the covariance matrices derived from rs-fMRI. A key methodological principle of this work is that the covariance structure of both the Hilbert envelopes of the EEG signal and the fMRI signal lie on a hypercone of symmetric positive matrices (SPD). In this manifold, the geodesic distance between covariance matrices can be estimated precisely. This provides a principled way of comparing multimodal weighted whole-brain networks/graphs within and across subjects.

Statistical inference has been shown to be a useful tool in examining the relationship between brain connectivity variables because it establishes a link between different modalities and it allows the generalization of the results from a sample set to the general population (Deligianni et al., 2010, 2011b, 2013). We use statistical inference based on sparse Canonical Correlation Analysis (sCCA) (Witten et al., 2009a; Witten and Tibshirani, 2009b) to link EEG and fMRI rs connectomes. Subsequently, subject specific EEG connectomes can be predicted from previously unseen fMRI connectomes and vice-versa. The predicted and measured functional connectomes are compared based on their geodesic distance and a prediction error is estimated based on leave-one-out cross validation. This allows us to statistically assess the information context of fMRI and EEG brain connectomes across bands.

This approach provides a rigorous multivariate statistical framework to quantify the importance of each connection in maximizing the relationship between EEG and fMRI connectivity. To this end, we extend the sCCA framework with the principle of randomized Lasso (Meinshausen and Buhlmann, 2010) to identify the most prominent connections that contribute to this relationship. This assigns a probability to each connection to be selected, and it offers a principled way to control for false positives. The sCCA loadings provide a data-driven weighting that minimizes the influence of noisy and unrelated connections, which do not contribute to the relationship between EEG and fMRI. This also provides a quantitative assessment of the overall accuracy of source localization in deep-gray matter regions.

## 2. Materials and methods

### 2.1. Imaging

Simultaneous resting-state EEG-fMRI was acquired from 17 adult volunteers (11 males, 6 females, mean age: 32.84 ± 8.13 years). The subjects had their eyes open and were asked to remain awake and fixate on a white cross presented on a black background. Subjects were asked to remain still and their head was immobilized using a vacuum cushion during scanning. Scalp EEG was recorded during the MRI scanning using a 64 channel MR-compatible electrode cap (BrainCap MR, Gilching, Germany) at native frequency of 1000 Hz. The electrodes were arranged according to the modified combinatorial nomenclature, referenced to FCz electrode. The electrocardiogram (ECG) was recorded, and EEG and MR scanner clocks were synchronized. Imaging data was acquired in a Siemens Avanto 1.5 T clinical scanner using a self-shielded gradient set with maximum gradient amplitude of 40 mTm^−1^ and standard 12 channel head receiver coil. Resting-state fMRI data were acquired based on a T2^*^-weighted gradient-echo EPI sequence with 300 volumes, *TR*/*TE* = 2160/30 ms, 30 slices with thickness 3.0 mm (1 mm gap), effective voxel size 3.3 × 3.3 × 4.0 mm, flip angle 75°, FOV 210 × 210 × 120 mm. A T1-weighted structural image was also obtained. Ethical approval has been obtained from the UCL Research Ethics Committee (project ID:4290/001) and informed consent has been obtained from all subjects.

### 2.2. Preprocessing

T1-weighted images were processed with Freesurfer to obtain gray matter (GM) 68 cortical regions and 14 subcortical regions (Desikan et al., 2006) (Table S1). Comparisons between two networks are easier to interpret when both are derived from the same set of nodes. Atlas-based parcellation allowed us to define corresponding nodes in both fMRI and the source-localized EEG signal. We propagate the anatomical labels from T1 space to native fMRI space using affine registration (Modat et al., 2010) to avoid erroneous warping of the image due to the drop out of gradient echo EPI images that result from local magnetic susceptibility effects. Anatomical labels are also propagated to MNI space, for the analysis of EEG, using non-rigid registration (Modat et al., 2010).

The first five volumes of rs-fMRI data are removed to avoid T1 effects and preprocessing of the functional data involves motion correction, high pass filtering (0.01 Hz) and spatial smoothing (5 mm) with FSL (Smith et al., 2004). To construct corresponding functional networks the fMRI signal is averaged across voxels within each GM ROI derived from the parcellation. The signal in WM and cerebrospinal fluid (CSF) is also averaged and along with the six motion parameters provided from FSL is linearly regressed out from the averaged time-series within each GM ROI.

EEG was corrected offline for scanner (Allen et al., 2000) and cardiac pulse related artifacts (Allen et al., 1998) using Brain Vision Analyzer 2 (Brain Products, Gilching, Germany). Subsequently, it was down-sampled to 250 Hz and exported to a standard binary format, which is supported by SPM12b (www.fil.ion.ucl.ak.uk) (Friston, 2007). The pre-processed EEG signal was also visually reviewed and noisy channels due to low impedances (≤100 kOhm) were excluded from the main analysis.

### 2.3. Analyses of the EEG signal

**Figure 1:**
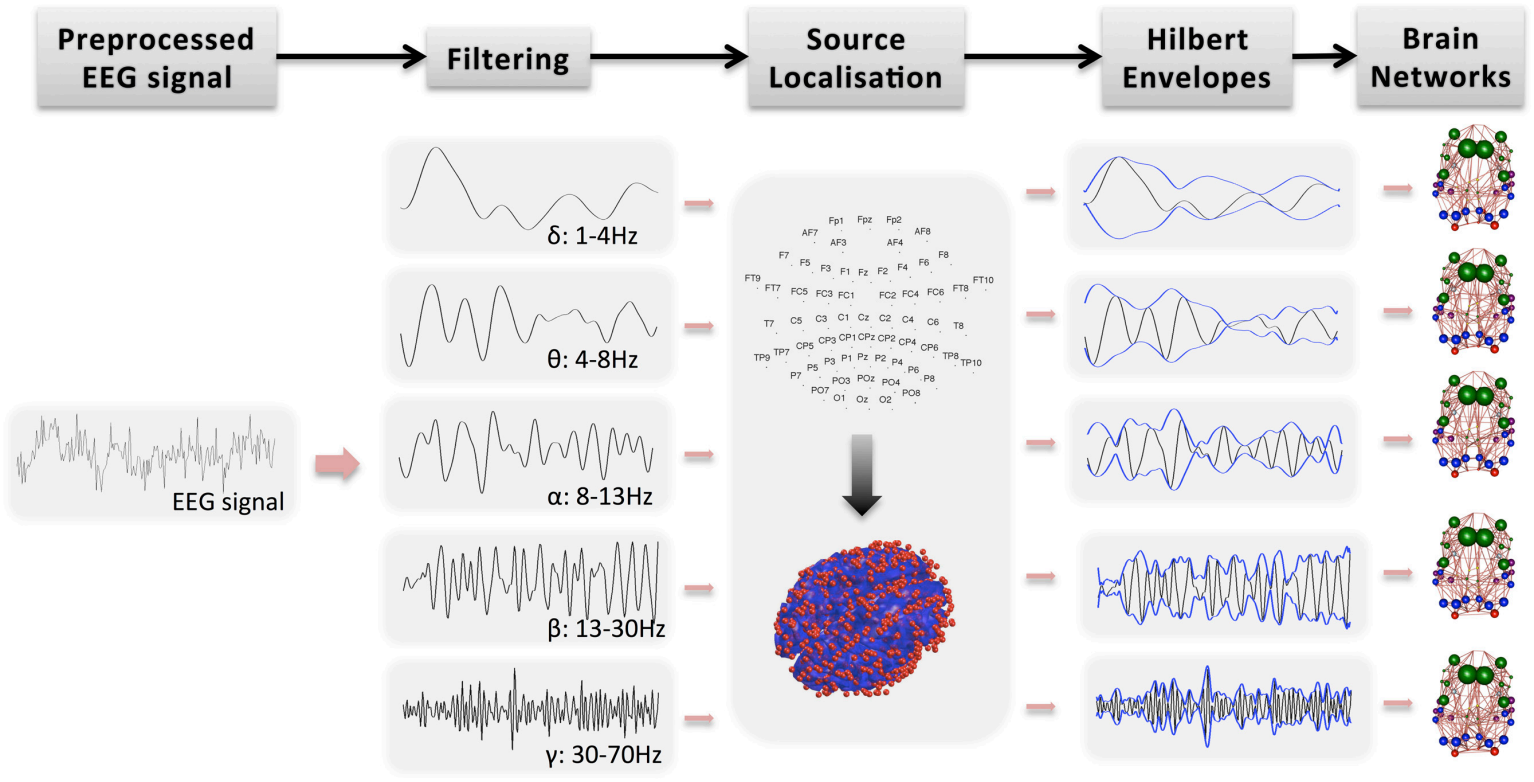
**Main steps toward deriving functional brain networks from the preprocessed EEG signal**.

Further analysis of the EEG signal is carried out with SPM12b. This involves the following steps also shown in Figure 1:

- Bandpass filtering: The signal is filtered into five bands: δ (1–4 Hz), θ (4–8 Hz), α (8–13 Hz), β (13–30 Hz), and γ (30–70 Hz). Phase delays are minimized by using zero-phase forward and reverse second order butterworth filter. Note that band-pass filtering is performed prior to source localization. Spatial resolution in beamforming is data dependent and thus it exhibits frequency dependent and time-variant magnitude characteristics (Barnes and Hillebrand, 2003). Traditional beamforming methods focus on narrow band signals because they approximate frequency independent of spatial selectivity.
- Segmentation into epochs: The signal is segmented into (fMRI) TR epochs (2.16 s).
- Definition of a head model: The standard template head model in SPM is used and the electrode positions are spatially transformed to match the template head. This provide reasonable co-registration of the original sensor positions to the MNI coordinate system of the template structural MRI image, even if individual subjects heads are considerably different from the template.
- Definition of forward model: The three-shell boundary element method (BEM) model is used for forward modeling and the lead fields are estimated using the Sarvas formulas for each point on the canonical cortical mesh.
- Source localization: EEG data is projected into source space using beamforming as implemented in SPM12b (Brookes et al., 2011a, 2012). Source localization allows spatial correspondence across subjects and modalities. It has also the potential to remove signal artifacts, which cannot be explained by the scalar beamformer. For each GM cortical region, the EEG signal is projected from sensor space to points randomly drawn from the region, independently for each subject. The region's center is always included whereas the number of points is proportional to region's volume. In Figure 1, the red dots on the 3D head model indicate the true density of random points drawn in cerebral cortex, which is around 0.7 points/cm^3^. These points have been picked randomly for each subject. Note that this approach of projecting the encephalography signal to specific brain locations has been used before to estimate thalamo-cortical coupling in MEG (Roux et al., 2013).
- Estimation of Hilbert envelope: We use two approaches to estimate the EEG time series and we produce results independently for each case: (a) We estimate the Hilbert transform across the whole down-sampled time series (WTS). Therefore, connectivity matrices are estimated based on the down-sampled time resolution of the EEG signal. (b) The EEG time-series are estimated as the average of the absolute value of the Hilbert transform within each epoch (AWE). This results in EEG time-series with corresponding time samples to the fMRI time-series. This approach provided the best agreement with the fMRI signal in Brookes et al. (2011a).
- Region average: Finally, within each region the Hilbert-transformed, source localized signal is averaged across the randomly distributed voxels to produce an EEG time-series per region. Note that similarly to the fMRI preprocessing, the first five epochs are not included in the average.

### 2.4. Estimation of functional brain connectomes

Once both EEG and fMRI average time-series have been estimated for each cortical region, we seek to derive the covariance structure of these signals. This assumes that the brain activity patterns are described by a Gaussian multidimensional stationary process. In this case, the covariance matrix characterizes fully the statistical dependencies among the underlying signals (Sporns et al., 2000). We use the inverse covariance, normalized to unit diagonal to characterize functional connectivity. The inverse covariance, also called the precision matrix, is directly related to partial correlation, which provides a measure of connectivity strength between two regions once the influence of the others has been regressed out. The correlation coefficient cannot distinguish between a direct signal transfer from node A–C from a signal transfer through other nodes, as for example from A to B to C. Partial correlation is the simplest approach in estimating direct connections. Furthermore, it offers a reasonable approximation of network structure for a scale of networks of up to few hundred of nodes, which is what is used in practice. This simplifies the problem of associating EEG with fMRI brain connectivity. Hence, there is no need to consider indirect signal transfer from one region to another via others (Deligianni et al., 2011a). To produce a well-conditioned, symmetric positive definite, (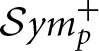), sample covariance matrix we use the shrinkage estimator (Krämer et al., 2009):

(1) $${\hat{\Sigma }}_{\lambda }=\lambda \hat{T}+(1-\lambda )\hat{\Sigma }$$

where the sample covariance matrix ${\hat{\Sigma }}_{\lambda }$ is estimated as a convex linear combination of the unrestricted sample covariance matrix $\hat{\Sigma }$ and the estimator $\hat{T}$, which is the identity matrix **I**. In this case, the optimal regularization parameter λ ∈ [0, 1] is determined analytically based on the Ledoit-Wolf theorem (Ledoit and Wolf, 2004). This approach provides a systematic way to regularize the sample covariance matrix and it has been shown to greatly enhance inference of gene association networks (Schäfer and Strimmer, 2005), where the number of variables *n* is much greater than the number of observations *p*. This approach allows one to estimate a well-conditioned covariance structure even when the number of connections grow quadratically with the number of ROIs without any prior information.

### 2.5. Predictive model based on sparse canonical correlation analysis (sCCA)

Canonical correlation analysis (CCA) is generally applied when one set of predictor variables **X** is to be related to another set of predicted variables **Y** and observations are available for both groups. Note that CCA is designed to deal with situations where the underlying variables are not statistically independent and, hence, they are inherently inter-correlated. The ultimate goal of CCA is to find two basis vectors (canonical vectors) *u, v*, one for each variable, so that the projections of **X**, **Y** onto these vectors, respectively are maximally linearly correlated.

In CCA all variables from both sets are included in the fitted canonical vectors. However, for the purpose of studying brain connectivity, we are interested in sparse sets of associated variables that would result in simultaneous multivariate dimensionality reduction and selection of the most relevant connections. Furthermore, it allows the emergence of interpretable links between EEG and fMRI connectivity data. Hence, we adapt sparse canonical correlation analysis (sCCA) to optimize the CCA criterion, subject to certain constrains (Witten and Tibshirani, 2009b):

(2) $$\begin{array}{c} {\text{maximize}}_{\text{u},\text{v}}u^{T}X^{T}Yv\\ \text{subject to}:\|u{\|}^{2}\leq 1,\;\|v{\|}^{2}\leq 1,\;\|u{\|}_{1}\leq c_{1},\;\|v{\|}_{1}\leq c_{2} \end{array}$$

‖*u*‖_1_ ≤ *c*_1_ and ‖*v*‖_1_ ≤ *c*_2_ represent the *L*_1_ (or *lasso*) penalty and they result in sparse canonical vectors *u, v* when the sparsity parameters *c*_1_ and *c*_2_, respectively, are chosen appropriately. Note that with *u* fixed, the criterion in Equation 2 is convex in *v*, and with *v* fixed, it is convex in *u*. Therefore, the objective function of this biconvex criterion increases in each step of an iterative algorithm (Witten and Tibshirani, 2009b):

(3) $$\begin{array}{c} u\leftarrow {\text{argmax}}_{\text{u}}u^{T}X^{T}Yv\text{ subject to}:\;\|u{\|}^{2}\leq 1,\;\|u{\|}_{1}\leq c_{1}\\ v\leftarrow {\text{argmax}}_{\text{v}}u^{T}X^{T}Yv\text{ subject to}:\;\|v{\|}^{2}\leq 1,\;\|v{\|}_{1}\leq c_{2} \end{array}$$

Here, we are interested in quantifying how well functional connectivity measured with EEG in different bands can predict fMRI brain connectivity and vice-versa. We use leave-one-out cross validation and thus for each subject *s* = 1, …, *S*, the sCCA model is trained based on the remaining *S* − 1 datasets. The number of components is estimated as the minimum of the ranks of the predictor and predicted variables in CCA. The penalty values *c*_1_, *c*_2_ are optimized in each cross-validation loop using an approach that permutes the rows of both the predictor and predicted variables of the sCCA (Witten and Tibshirani, 2009b). Optimization takes place with exhaustive search on a grid of values.

Subsequently, a subject-specific rs-fMRI connectome **Y_s_** is predicted from its previously unseen EEG connectome **X_s_** according to:

(4) $${\hat{Y}}_{s}={\left(uX_{s}\right)}^{+}Dv^{+}$$

Vice-versa a subject-specific EEG **X_s_** conenctome can be predicted from its rs-fMRI connectome **Y_s_** based on the same sCCA solution of *u* and *v* vectors:

(5) $${\hat{X}}_{s}={\left({\left(Y_{s}v\right)}^{⊤}\right)}^{+}Du^{+}$$

**D** is a diagonal matrix with the canonical correlation scores and ^+^ denotes the pseudoinverse. The sCCA optimization problem being solved is symmetric in the two variables. However, the algorithm finds a local optimum, by first updating one, then updating the other criterion. Therefore, depending on the order of updates, the local optimum obtained might be different. We found that there was no practical difference when we reverse the optimization approach.

Here, both **X** and **Y** are matrices with rows the vectorized upper or lower triangular part of the precision matrices across subjects. The diagonal elements of the normalized precision matrix are excluded since they are always ones. CCA applies to these elements without any further restrictions and hence there is no explicit guarantee the predicted precision matrix would be SPD.

### 2.6. A metric to compare covariance matrices

We are interested in estimating the similarity between predicted and estimated connectivity matrices based on a distance metric that quantifies differences in the space of covariance matrices. Precision and covariance matrices lie in the space of symmetric definite positive matrices 
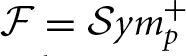
. The standard Euclidean distance on matrices, the Frobenius norm, does not account for the geometry of this space. Thus, this norm is ill-suited to quantify prediction errors. However, 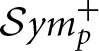 can be parameterized as a Riemannian manifold using an intrinsic metric (Förstner and Moonen, 1999; Arsigny et al., 2006):

(6) $$d_{AI}{\left(P,G\right)}^{2}=tr{\left(\log G^{-\frac{1}{2}}PG^{-\frac{1}{2}}\right)}^{2}$$

This metric has been used successfully to build statistical frameworks of precision matrices 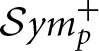 (Deligianni et al., 2011b). *d*_*AI*_ is a distance metric, invariant to affine transformations and inversion, appropriate to quantify the distance between covariance matrices from biological data successfully (Mitteroecker and Bookstein, 2009).

The *d*_*AI*_ measure is applied in a leave-one-out cross-validation loop outside the sCCA algorithm to reliably estimate the out-of-sample modeling error. We have shown before that the *d*_*AI*_ metric is suitable in quantifying the loss in a structured-output multivariate regression predictive framework, because it accounts for the geometry of the output space, and it demonstrates evidence of statistical consistency (Deligianni et al., 2013). Since CCA is closely related to multivariate multiple regression analysis (Lutz and Eckert, 1994), we argue that *d*_*AI*_ is appropriate to compare the prediction performance of different functional models of brain connectivity.

### 2.7. Identification of relevant connections

It is of great interest to identify which rs-fMRI connections are mostly related to functional connections derived in each EEG band. Toward this objective we concatenate the connections across all EEG bands to one variable $\overset{⌣}{X}$, whereas the rs-fMRI connectivity variable remains the same **Y**. We are interested in applying the same biconvex criterion described in Equation 3 to solve the sCCA problem that aims to find the parameters that maximize the linear relationship between $\overset{⌣}{X}$ and **Y**, Equation 2. The concatenation of the connections across all EEG bands is advantageous because it does not require the choice of a sparsity parameter for each band independently, which would hinder meaningful comparisons across bands.

Subsequently, we modify the biconvex criterion in sCCA, Equation 3, based on the randomized Lasso principle (Meinshausen and Buhlmann, 2010). Therefore, Equation 3 takes the following form:

(7) $$\begin{array}{c} u\leftarrow {\text{argmax}}_{\text{u}}{\left(w_{x}\cdot u\right)}^{T}{\overset{⌣}{X}}^{T}Yv\text{ subject to}:\\ \;\|u{\|}^{2}\leq 1,\;\|u{\|}_{1}\leq c_{1},\;w_{x}\in \{1,0.5\}\\ v\leftarrow {\text{argmax}}_{\text{v}}u^{T}{\overset{⌣}{X}}^{T}Y(v\cdot w_{y})\text{ subject to}:\\ \;\|v{\|}^{2}\leq 1,\;\|v{\|}_{1}\leq c_{2},\;w_{y}\in \{1,0.5\} \end{array}$$

*w*_**x**_ and *w*_**x**_ are the coefficients weights chosen randomly equal to 0.5 or 1, as recommended by Meinshausen and Buhlmann (2010); Deligianni et al. (2013). The randomized sCCA criterion in Equation 7 is optimized several times, which is effectively a strategy of resampling the connectivity data. Note that *c*_1_ and *c*_2_ was chosen initially based on a permutation strategy and they remain the same through out the randomized Lasso iterations. The probability of selecting a connection is then given by the number of times the coefficient is selected over the number of repetitions. This provides a principled control on thresholding false positives and it is a significant improvement over the standard Lasso penalization, which does not provide any information on the statistical significance of the selected features. Another important benefit of the randomized Lasso is that it decreases the dependence of the selected coefficients on the initial choice of the sparsity parameter, *c*_1_ and *c*_2_.

## 3. Results

We present results based on brain connectomes derived from the whole time series of EEG (WTS) as well as corresponding results derived based on brain connectomes estimated from averaging the Hilbert transformed EEG signal within each epoch (AWE).

**Figure 2:**
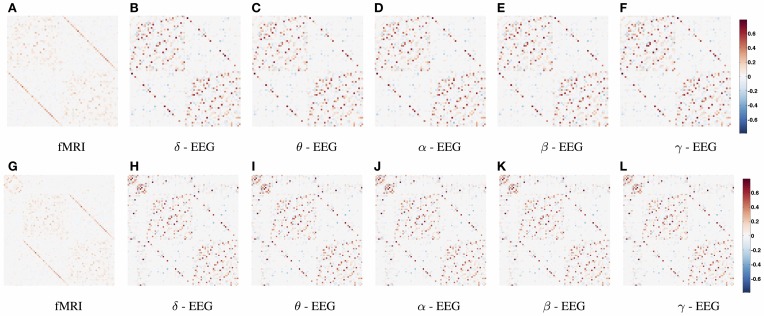
**Average functional connectivity matrices across subjects in fMRI and EEG δ, θ, α, β, and γ bands, respectively**. In both fMRI and EEG, the precision matrix has been estimated across the whole time samples (WTS). **(A–F)** Depicts the partial correlation within cortical regions (68 × 68), whereas the bottom row demonstrates the partial correlation within cortical and subcortical regions (82 × 82). (The regions are given in Table S1 and they are ordered similarly in their matrix representation). For the cortical regions, the top left matrix quadrant represents connections within left hemisphere (lh), the bottom right represent connections within the right hemisphere (rh), and the remaining quadrants represent inter-hemispheric connections. At **(G–L)**, the subcortical regions have been added at the top left corner of the connectivity matrices.

In Figure 2 we show the average functional connectivity matrices across subjects in fMRI and EEG δ, θ, α, β, and γ bands, respectively. In both fMRI and EEG, the precision matrices have been estimated based on time-series across the whole experiment (WTS). Matrices are symmetric, since they reflect correlation and this implies that there is no directionality information. Each of the connectivity matrices has been estimated by averaging (mean) each connection across all subjects. All matrices have two distinctive parallel lines to the diagonal that represent homologous inter-hemispheric connections. These are strong in both fMRI and EEG across all bands, whereas in EEG we also observe strong intra-hemispheric connections. The top row depicts the partial correlation within cortical regions and results in 68 × 68 matrices. The bottom row demonstrates the partial correlation within cortical and subcortical regions and results in 82 × 82 matrices. For the cortical regions, the top left matrix quadrant represents connections within the left hemisphere (lh), the bottom right represent connections within the right hemisphere (rh) and the remaining quadrants represent inter-hemispheric connections. In the bottom row, the subcortical regions have been added at the top left corner of the connectivity matrices. (The regions are given in Table S1 and they are ordered similarly in their matrix representation.)

**Figure 3:**
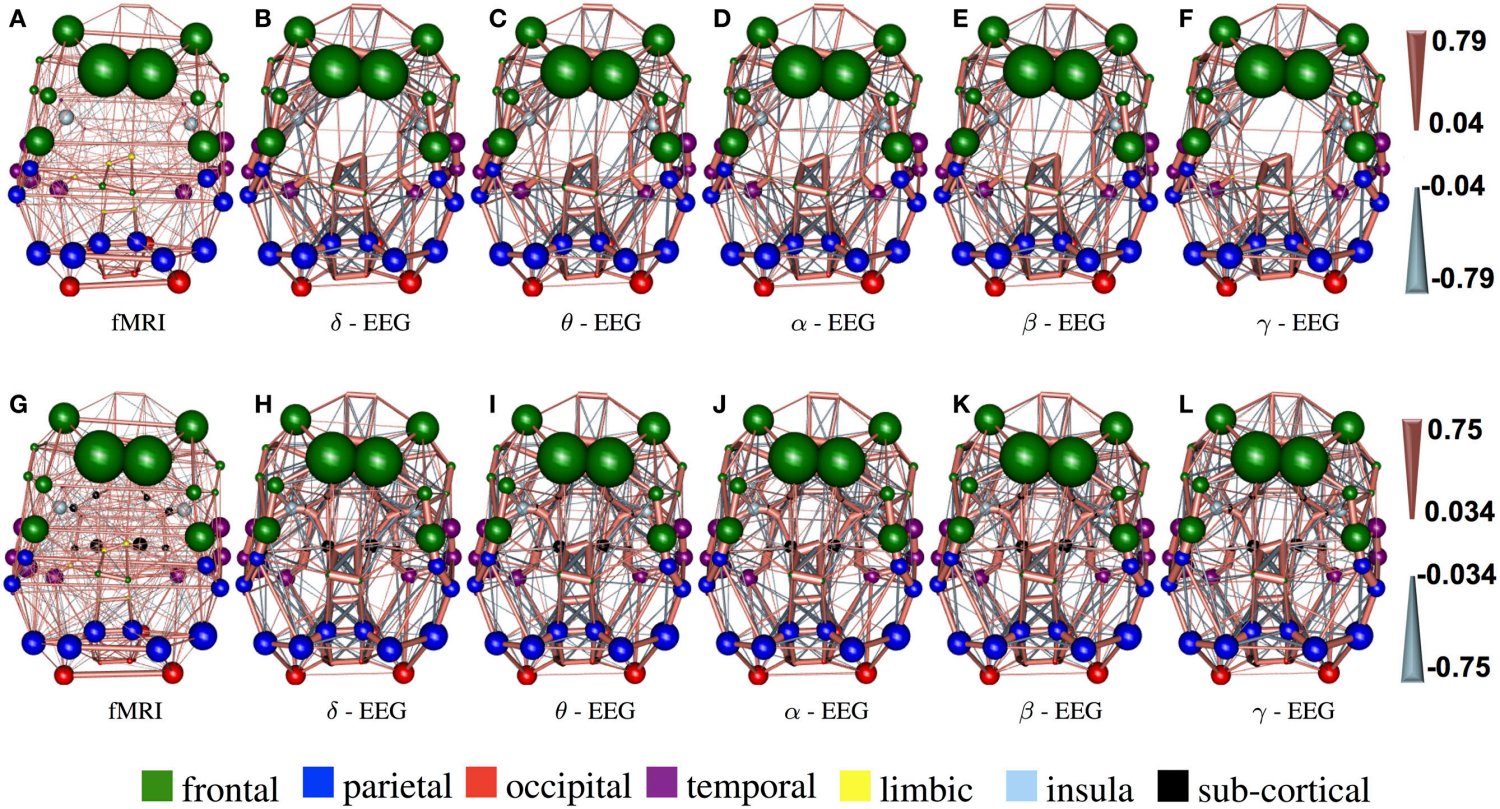
**The connections with the 15% highest absolute value in Figure 2 (WTS) are shown as 3D graphs in standard space**. Connections are represented as cylinders with salmon color when they are positive and slate-gray when they are negative. Brain regions are represented with spheres. Their centers and radii represent the centers of mass of each underlying region and its volume, respectively. The color-coding corresponds to different parts of the brain. **(A–F)** Shows partial correlation networks within cortical regions, whereas **(G–L)** shows partial correlation networks within cortical and subcortical regions.

In Figure 3, the connections with the 15% highest absolute value in Figure 2 (WTS) are shown as 3D graphs in MNI space. The top row shows partial correlation networks within cortical regions, whereas the bottom row shows partial correlation networks within cortical and subcortical regions. In rs-fMRI connectomes inter-hemispheric connections dominate, whereas across connectomes from each EEG band intra-hemispheric connections are predominant. In particular, brain regions are represented with spheres. Their centers and radii represent the center of mass of each underlying region and its volume, respectively. The color-coding of the spheres corresponds to different parts/lobes of the brain. Connections above the 15% threshold are represented as cylinders with salmon color when they are positive and slate-gray when they are negative. The diameter of the cylinder is proportional to the connection's strength, scaled independently in the fMRI connectome and the connectome from each EEG band.

**Figure 4:**
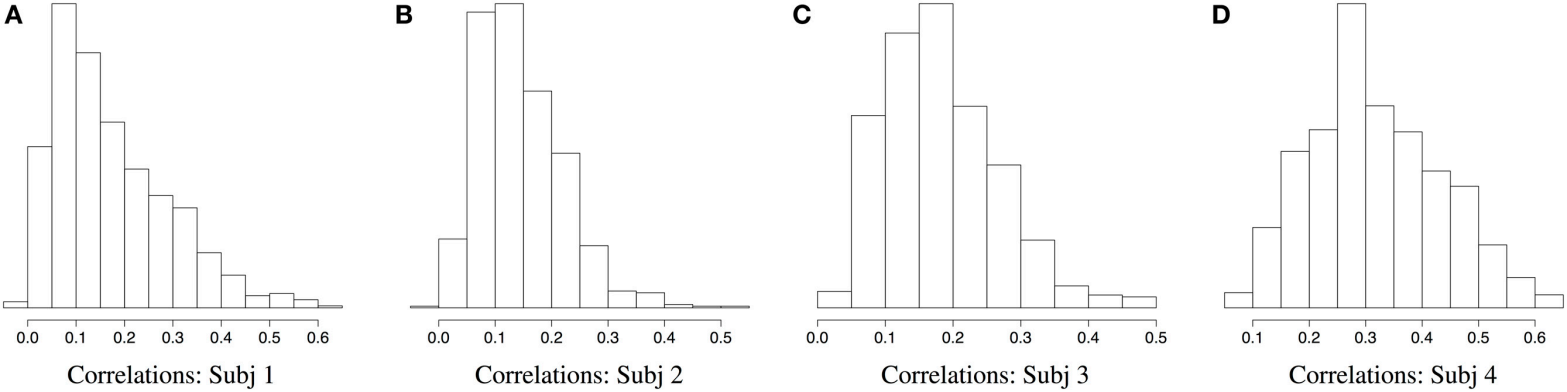
**Histograms of correlations between bands across all cortical ROIs for four subjects**.

Figures 2, 3 demonstrate a relatively similar covariance structure across the EEG frequency bands. To examine whether there is a broadband phenomenon where all frequency bands fluctuate together within ROIs, or whether they are minimally correlated, we plot the histograms of correlations for four subjects in Figure 4. Within each ROI, we estimated the correlation matrix (5-by-5) of the averaged time series (WTS) for each band. Here, we show the histograms of the off diagonal correlation elements across all cortical ROIs. These results show low correlation values between bands and demonstrate that the whole-brain EEG connectomes are not driven by broadband signal changes but rather EEG signals at different frequencies operate within the same networks.

**Figure 5:**
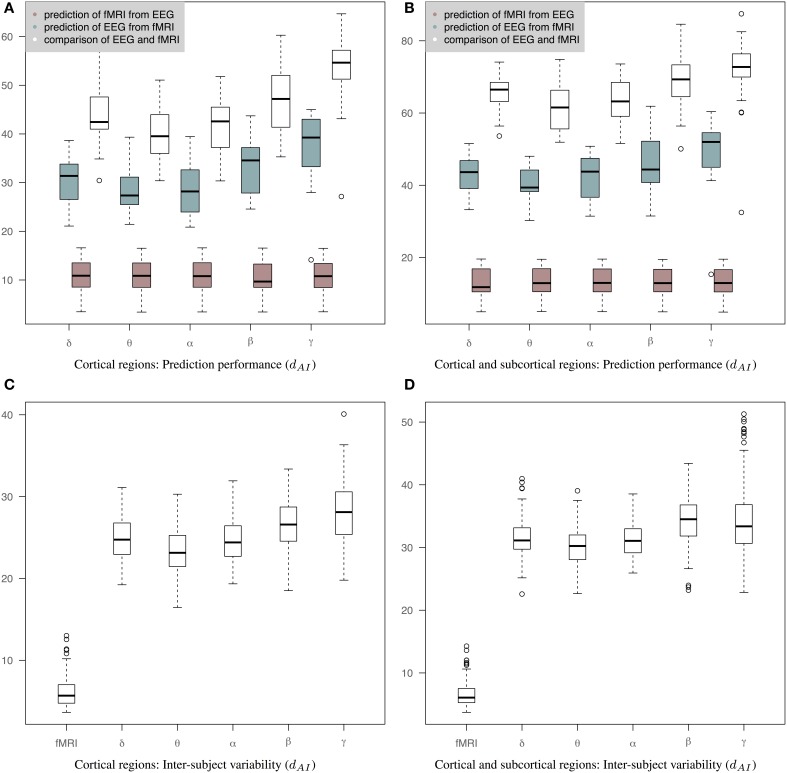
**Results of prediction performance (WTS)**. This figure presents results of prediction performance and inter-subject variability when both fMRI and EEG precision matrices are estimated based on all time samples. The distance between the rs-fMRI precision matrices and each of the EEG frequency banded precision matrices estimated with *d*_*AI*_. The smaller the distance the more similar the connectivity matrices should be. **(A)** It shows results based only on cortical regions that summarize the prediction performance of fMRI from EEG (brown box-plots) and vice-versa (green box-plots) across bands, as well as the distance between the fMRI precision matrices and the EEG precision matrices within subjects (white box-plots), **(B)** It shows results based on both cortical and sub-cortical regions that summarize the prediction performance of fMRI from EEG (brown box-plots) and EEG from fMRI (green box-plots) across bands, as well as the distance between the fMRI precision matrices and the EEG precision matrices within subjects (white box-plots), **(C)** It shows inter-subject variability for the precision matrices estimated within cortical regions. **(D)** It shows inter-subject variability for the precision matrices estimated within cortical and subcortical regions.

Figure 5 shows results of prediction performance and inter-subject variability for the case of precision matrices derived based on the WTS approach. Results demonstrate that sCCA has improved the agreement between the predicted connectivity matrices and the corresponding measured connectivity matrices. Note that the optimization objective of Equations 2, 3 does not optimize the distance between connectivity matrices directly. sCCA learns the relationship between EEG and fMRI connections across subjects and as a result the Euclidean distance between the predicted and measured connectomes is minimized. This usually results in minimizing the geodesic distance between connectomes too. The prediction performance are represented based on the *d*_*AI*_ metric, which reflects geodesic distance between SPD matrices. The smaller the distance the more similar the connectivity matrices should be and subsequently the better the performance of the sCCA training. Figure 5A shows results based only on cortical regions that summarize the prediction performance of fMRI from EEG (brown box-plots), EEG from fMRI (green box-plots) across bands, as well as the distance between the fMRI precision matrices and the EEG precision matrices within subjects (white box-plots). Figure 5B shows similar results based on both cortical and sub-cortical regions. In all cases, the performance of the predictions is estimated based on leave-one-out cross validation. *c*_1_ and *c*_2_ have been optimized in each cross-validation loop according to a permutation-based algorithm [6]. The number of components is estimated as the minimum of the ranks of the variables *X* and *Y*.

The ability to predict a rs-fMRI precision matrix from an EEG precision matrix remains relatively similar across bands and it is substantially better than predicting an EEG connectivity matrix from a rs-fMRI precision matrix. This is also shown with a Wilcoxon rank-sum test, which demonstrates significant statistical differences between the prediction performance of EEG from fMRI and the prediction performance of fMRI from EEG across all bands (*p*-values < 1e-05). On the contrary, the prediction of EEG from fMRI is considerably modulated across bands with the low frequency bands (δ, θ, α) performing better, similarly to the within-subject distance between the measured fMRI and EEG connectomes. In Table 1 we show the *p*-values of Wilcoxon rank-sum tests for assessing differences between intra-subject comparisons of EEG and fMRI connectomes across bands. Figures 5C,D shows inter-subject variability for the precision matrices estimated within only cortical and both cortical and subcortical regions, respectively. Inter-subject variability in fMRI is considerably lower than inter-subject variability across all EEG bands.

**Table 1:** ***P*-values of Wilcoxon rank-sum test for assessing differences between intra-subject comparisons of EEG and fMRI across bands (WTS) shown in Figure 5**.

	θ	α	β	γ
**(A) CORTICAL CONNECTOMES**				
δ	0.07	0.39	0.23	**3.4e-04**
θ		0.39	**0.006**	**1.2e-05**
α			**0.03**	**6.1e-05**
β				**9.4e-03**
**(B) CORTICO-SUBCORTICAL CONNECTOMES**				
δ	0.12	0.31	0.18	**0.002**
θ		0.88	**0.01**	**0.0004**
α			0.06	**0.003**
β				0.06

*Bold values indicate p < 0.05*.

**Figure 6:**
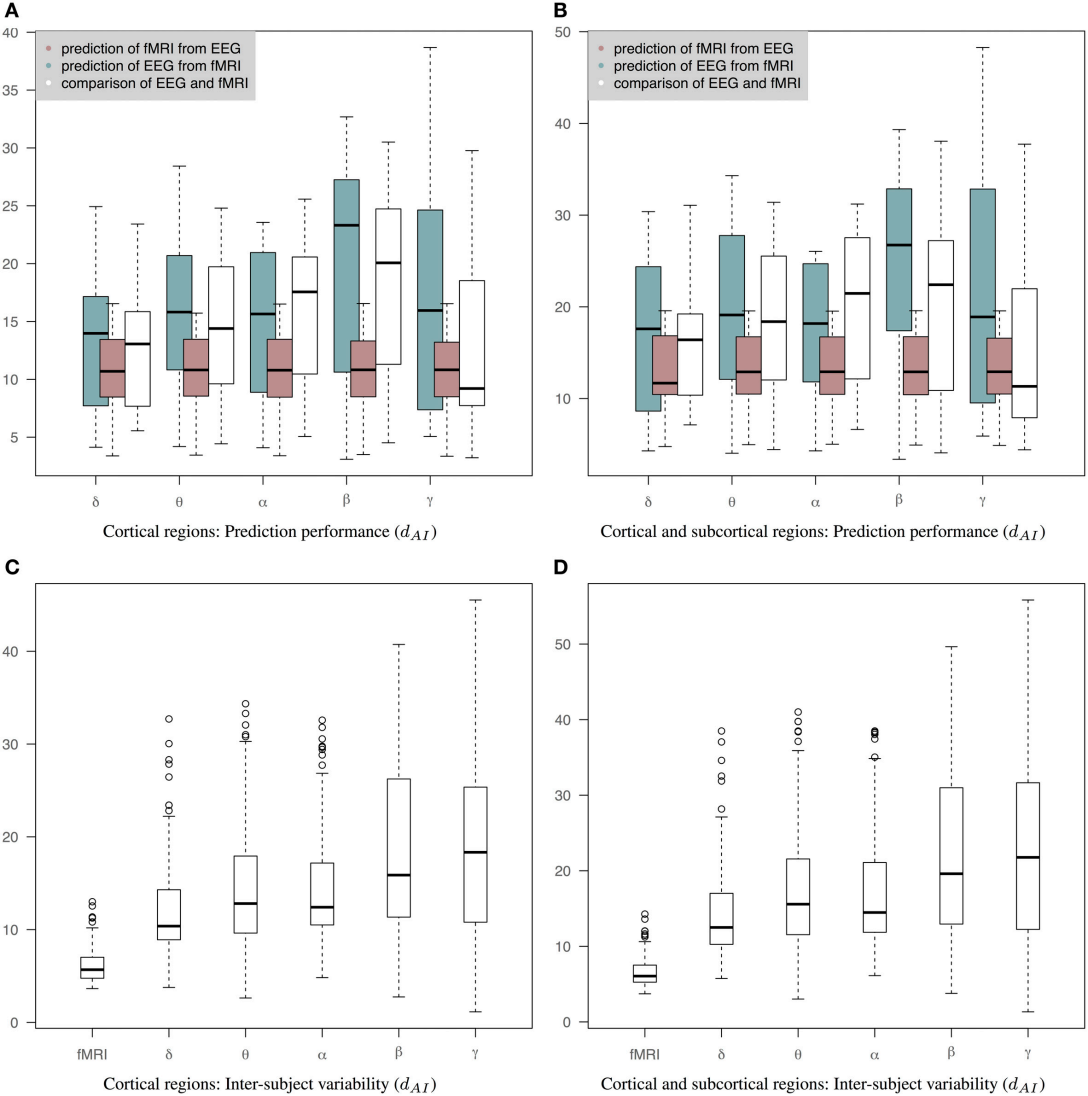
**Results of prediction performance (AWE)**. This figure presents the same results as Figure 5 but EEG time series have been averaged within each epoch, which is equal to the fMRI-TR. The distance between the rs-fMRI precision matrices and each of the EEG frequency banded precision matrices estimated with *d*_*AI*_. The smaller the distance the more similar the connectivity matrices should be. **(A)** It shows results based only on cortical regions that summarize the prediction performance of fMRI from EEG (brown box-plots) and vice-versa (green box-plots) across bands, as well as the distance between the fMRI precision matrices and the EEG precision matrices within subjects (white box-plots), **(B)** It shows results based on both cortical and sub-cortical regions that summarize the prediction performance of fMRI from EEG (brown box-plots) and EEG from fMRI (green box-plots) across bands, as well as the distance between the fMRI precision matrices and the EEG precision matrices within subjects (white box-plots), **(C)** It shows inter-subject variability for the precision matrices estimated within cortical regions. **(D)** It shows inter-subject variability for the precision matrices estimated within cortical and subcortical regions.

Figure 6 is similar to Figure 5 but the EEG connectivity matrices have been produced by averaging the Hilbert transformed signal within epochs (AWE). Therefore, each EEG time sample corresponds to a single fMRI time sample. (The corresponding connectivity matrices and 3D graphs are shown in Figures S1, S2.) In this case, the distance between fMRI and EEG is smaller across all bands compared to Figures 5A,B. Nevertheless, the prediction of fMRI from EEG is better than the prediction of EEG from fMRI. A Wilcoxon rank-sum test shows significant statistical differences in θ and β bands with *p*-values of 0.04 and 0.01, respectively, for cortical connectomes and *p*-values of 0.04 and 0.005 for cortico-subcortical connectomes. sCCA training does not improve the performance of predicting EEG from fMRI compared to the original within-subject distance of fMRI and EEG connectivity. This may reflect the limits of the sCCA since *d*_*AI*_ is not optimized explicitly and the original distance of the connectivity matrices is already low. Figures 6C,D show inter-subject variability for only cortical and both cortical and subcortical regions, respectively.

**Figure 7:**
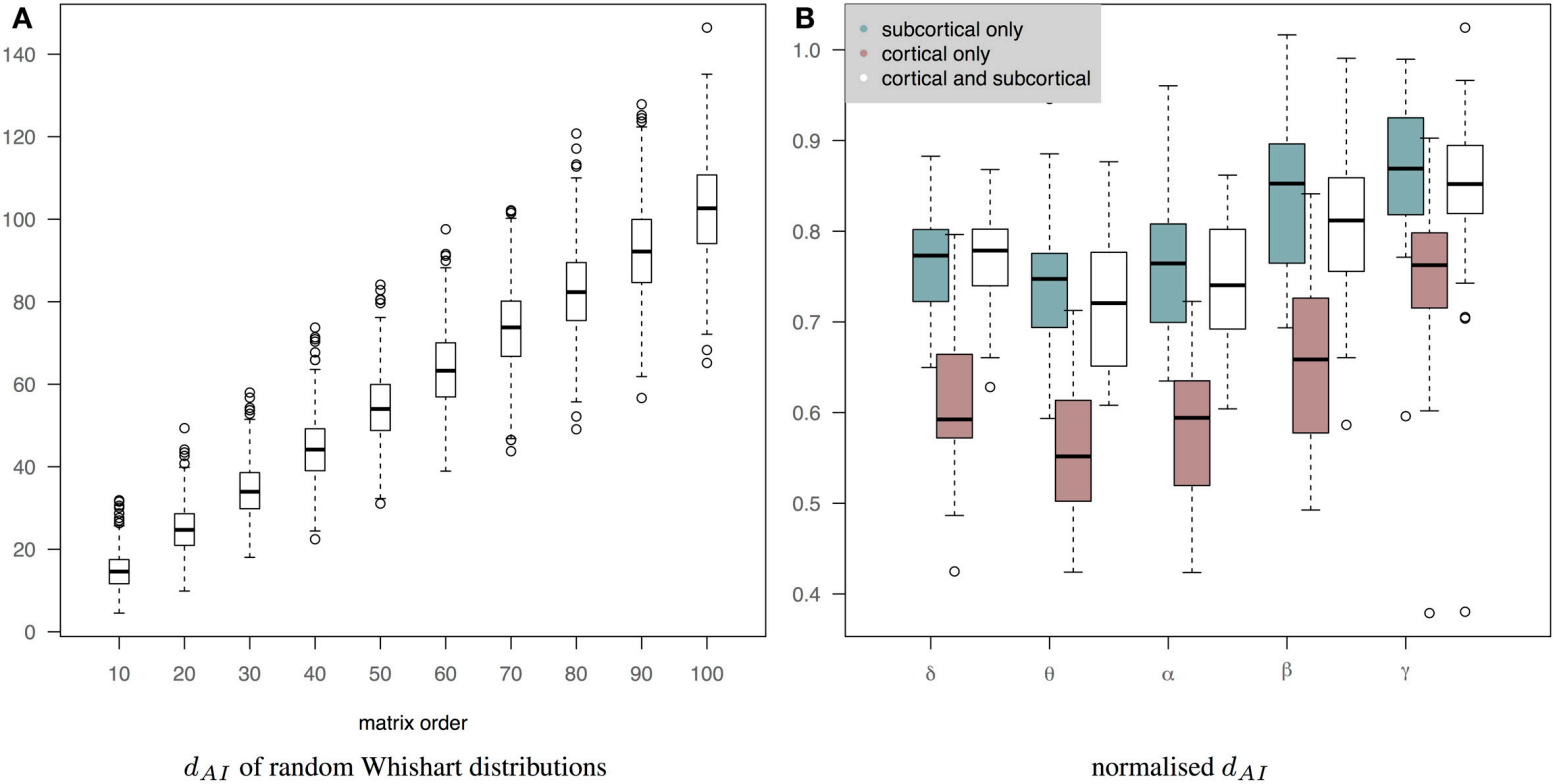
**(A)** It shows how the *d*_*AI*_ metric scales with the number of regions represented in the precision matrices. 1000 pairs of precision matrices were drawn from random Whishart distributions from matrix order of 10–100. Subsequently, the distance between the pair of matrices was estimated based on the *d*_*AI*_ metric. **(B)** It shows the normalized distance between the measured EEG and fMRI precision matrices across bands when we use only subcortical structures, only cortical structures and both cortical and subcortical structures. *d*_*AI*_ has been normalized based on the line fit of the median values of the simulation data. This is approximately equivalent of dividing by the number of regions.

Figure 7B shows the normalized distance between the measured EEG and fMRI precision matrices across bands when we use only subcortical structures, only cortical structures and both cortical and subcortical structures. The inclusion of the subcortical regions in the connectome increases the within subject distance between EEG and fMRI matrices (less similar connectomes). Several factors can account for this finding, including, the limitation of EEG source reconstruction in deep brain structures. Note that *d*_*AI*_ has been normalized based on the line fit of the median values of the simulation data in Figure 7A. This is approximately equivalent of dividing by the number of regions. Figure 7A shows how the *d*_*AI*_ metric scales with the number of regions represented in the precision matrices. Simulation results come from the comparison of 1000 pairs of precision matrices drawn from random Whishart distributions of matrix order from 10 to 100. To investigate further whether incorporating subcortical regions improves the prediction performance, we examined the performance of prediction of cortical fMRI connectomes from EEG cortical and cortico-subcortical connectomes. In this case, the number of regions in the predicted connectomes is the same and there is no need for any normalization. Subsequently, we used a paired Wilcoxon test to examine significance in each band. Our results showed that there is a trend that cortico-subcortical EEG connectomes predict cortical fMRI connectomes better than using cortical EEG connectomes alone. This difference is significant in the δ band (*p* = 0.01) and close to significance in the θ band (*p* = 0.08).

**Figure 8:**
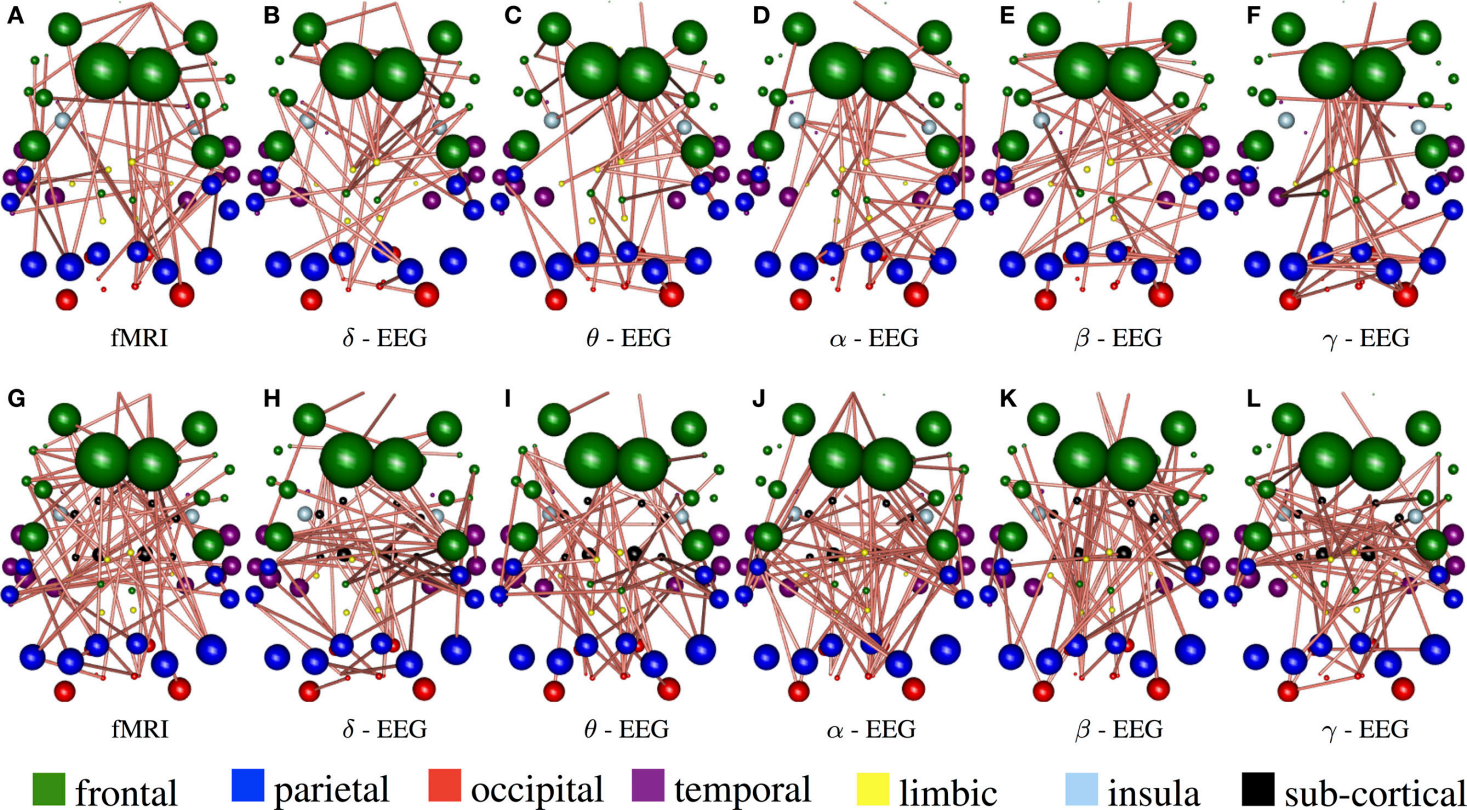
**Results derived from randomized Lasso for the WTS case**. These reflect the 2% connections selected more often over all sCCA repetitions. **(A–F)** Shows results with the precision matrices derived based on cortical regions only, whereas **(G–L)** shows the results obtained with precision matrices that include both cortical and subcortical regions.

Figure 8 demonstrate the results of 98050 randomized Lasso iterations for the EEG brain networks estimated based on WTS. (Figure S3 shows the corresponding results of the AWE case.) These results highlight the most prominent connections in sCCA from rs-fMRI (*v*) and EEG (*u*) brain connections across all bands. For this experiment we concatenate all the connections across all EEG bands to form the canonical variable $\overset{⌣}{X}$, whereas **Y** is the brain connectivity as it is measured from rs-fMRI. This allows us to draw the most relevant variables across all bands under the same sparsity parameters *c*_1_ and *c*_2_. Finally, we measure how many times each connection is selected out of the 98050 iterations and this provides us with a probability measure of confidence representing the importance of the underlying connection in maximizing the relationship between fMRI and EEG. The top row shows the 2% connections with the highest selection probability in fMRI and each EEG band for cortical regions only. The bottom row shows the 2% connections with the highest selection probability for the configuration with both cortical and subcortical regions. We note that the selected features are mostly long-range connections. To our knowledge, the results of randomized Lasso represent the first attempt to show inter-relations between EEG and fMRI whole-brain connectomes.

## 4. Discussion

We utilized the band-limited power envelope of the EEG signal to estimate an average time-series for each gray-matter cortical region based on a standard atlas-based parcellation. Based on this approach we describe functional connectivity with covariance matrices between corresponding regions across subjects and modalities. This allows us to compare resting-state functional connectivity derived across frequency bands from EEG with resting-state functional connectivity derived from BOLD fMRI. To our knowledge, we are the first to investigate the relationship between synchronous fMRI and EEG connectomes across frequency bands in a whole-brain, using source space analysis. fMRI connectivity is dominated by the inter-hemispheric connections between homologous areas, whereas brain connectivity derived from EEG shows a more complex pattern of connections composed by both intra-hemispheric and inter-hemispheric connections. We also observe that EEG connectomes in low frequency bands are the most similar to resting-state fMRI connectomes based on their geodesic distance of the underlying precision matrices.

One possibility is that the low frequency bands in EEG are most predictive due to their higher signal-to-noise ratio. However, low frequency bands are affected from small drifts, eye blinks, cardiac, and respiration cycle and so on, whereas muscle artifacts and channels with low impedance affect higher frequencies. In addition for EEG-fMRI this is additionally complicated by the gradient and pulse artifacts that provide sources of structured noise in particular in the alpha band at the slice frequency. Given this noise distribution, it is unlikely that the prediction difference of the EEG bands is driven by the signal noise differences between bands. Nevertheless, we cannot exclude the possibility that differences in SNR across frequencies could explain some of the differences in similarity between fMRI and EEG brain connectomes across bands.

Subsequently, we examine the connectivity derived from simultaneous EEG and fMRI by means of statistical prediction. An advantage of a predictive framework of EEG and fMRI connectomes is that it removes noise that it is present in one modality and not the other. We use sCCA to predict EEG brain connectivity from fMRI and vice-versa. To evaluate the prediction performance we use leave-one-out cross validation and we compare the predicted connectivity matrix with the observed connectivity matrix. We demonstrate that the performance of predicting fMRI connectivity from EEG is considerably better than predicting EEG from fMRI across all bands. In fact, the prediction performance of EEG from fMRI follows a similar pattern to the distance between the original precision matrices, whereas the prediction performance of EEG from fMRI is relatively stable across bands. There is no significant improvement in prediction of fMRI from EEG using the joint information across multiple EEG frequency bands. Note that increasing the number of variables does not necessarily increase the prediction performance, since we use cross-validation loop to control for over fitting.

This finding has several important implications. Firstly, it shows that there are signatures of rs-fMRI dynamics across EEG frequencies. This is consistent with the concept of nested oscillations and cross spectral coupling often found within EEG (Penny et al., 2008). Note that we have used envelope correlation amplitude and thus the phase information is not preserved. Nevertheless, if the phase-amplitude locking, which indicates nested oscillations, is intermittent then a large overall amplitude correlation is also expected (Penny et al., 2008). Secondly, it likely reflects the greater dynamic information content captured by EEG in this particular spatial scale. Although, the spatial resolution of source localization is in the scale of 1–2 cm, most fMRI network analysis studies involve averaging the hemodynamic signal within larger regions. Our results indicate that in this spatial resolution the information carried in the EEG signal is richer than the averaged hemodynamic activity. In this context, the question of which EEG band represents best the fMRI is not important; any EEG band can provide similar connectivity information. This implies that scalp EEG can be used to provide similar information to resting state fMRI based connectomes at substantially reduced cost while providing much greater possibilities in dynamic information content. This might be because of the coarse brain parcellation, which limits spatial resolution to the size of the underling cortical regions. However, most current fMRI studies tend to examine connectivity at this scale.

On the other hand, the inclusion of the subcortical regions results in more dissimilar fMRI and EEG connectomes even when we account for the difference in the number of regions. This may indicate that the highly complex cortico-subcortical interactions are not adequately captured with EEG alone. Cortico-subcortical interactions play an important role in regulating physiological rhythms that are associated with sleep or wakefulness, motor control and so on. Furthermore, they have an eminent role in pathological conditions such as the propagation of epileptic activity in several epilepsy syndromes (Kahane and Depaulis, 2010; Moeller et al., 2013). Therefore, further investigation on how multi-modal data can improve the sensitivity in detecting these interactions both in space and time is crucial in discovering new treatments and understanding how brain networks work. Our multi-modal connectivity analysis demonstrate evidence that incorporating sub-cortical structures in EEG connectomes improves the prediction of cortical fMRI connectomes. Therefore, a cutoff in weights might be appropriate in some circumstances, but due to the sparsity constraints a weight should only be large enough to be influential if the corresponding edge is genuinely informative for the prediction. It should therefore not be necessary to explicitly down-weight or ignore connections carrying little predictive information. We acknowledge that there is controversy in the ability to detect subcortical sources with EEG source imaging alone (Muthuraman et al., 2014). However, Muthuraman et al. also showed that sources in deep gray matter structures are present in EEG data when segments with higher SNR are selected indicating a lower sensitivity of EEG to detect deep-gray matter sources compared to MEG data. Nevertheless, Plomp et al. linked event related potentials recorded with EEG with sources in the insula and subcortical areas such as the parahippocampus and the thalamus (Plomp et al., 2010). Furthermore, Moeller et al. demonstrated the ability of electrical source imaging in identifying deep sources in the thalamus and in revealing similar neuronal networks as with simultaneously acquired fMRI (Moeller et al., 2013). In any case, as we have discussed here, there is evidence in the literature and in our data to suggest that there may be some information in the scalp EEG which is attributable to deep sources.

Furthermore, we showed that the connectomes derived in low frequency EEG bands (δ, θ, and α) resemble best rs-fMRI connectomes. This conclusion results from estimating the precision matrices over the whole down-sampled EEG Hilbert-transformed time-series (WTS). When connectivity is estimated based on the average of the signal envelope within epochs (AWE), the geodesic distance between EEG and fMRI connectomes is smaller, reflecting the fact that averaging the EEG signal within epochs of equal duration to fMRI TR, approximates the rs-fMRI signal better. Effectively, this reduces the information content in the EEG in a way that better resembles the fluctuations observed in the BOLD signal. Furthermore, this temporal averaging of the EEG means that the difference in prediction performance between bands is smaller than the inter-subject variability within-band.

In literature there is on-going controversy about which band in EEG mostly resembles rs-fMRI connectivity. Our results are consistent with de Pasquale et al. where a seed-based analysis was used to correlate the dorsal and default mode networks with spontaneous MEG activity (de Pasquale et al., 2010) and showed that the band limited MEG signal in theta, alpha and beta bands is primarily related to BOLD fMRI connectivity. Similarly, Brookes et al. observed higher spatial agreement between resting-state fMRI and MEG in the α and β bands (Brookes et al., 2011b). However, in de Pasquale et al.s MEG study inter hemispheric correlation between homologous regions was not observed in despite it being a typical feature of resting state fMRI studies, being observed in the first such study by Biswal et al. (1995). Furthermore, Cabral et al. found that the strength of correlation between brain regions peaks at the α and the lower end of the β frequency bands, both in MEG and in simulated connectivity based on coupled oscillators with parameters derived from structural networks (Cabral et al., 2014). On the other hand, work in anaesthetized rats suggested that in the fMRI signal is mostly correlated to δ band (epidural) electrophysiological measures (Lu et al., 2007), whereas Magri et al. highlighted α, β, and γ bands as mostly related to BOLD fMRI spontaneous activity in anaesthetized monkeys.

There is some consensus among studies that the best agreement between rs-fMRI and EEG signal is in the α frequency range. Our results also highlight low frequency bands, which could result from that both fMRI and EEG connectomes describe mostly long-distance connections due the relatively large volume of the underlying regions. In fact, evidence suggests that the more distant two neural assemblies are, the longer the signal-conduction delay between them. This biases the maintenance of a phase relationship between the two signals over long cortical distances to low frequencies (Schölvinck et al., 2013). Also small time shifts in high frequencies cause proportionally large phase shifts, which limits correlations in high frequencies. EEG and fMRI provide measurements of whole-brain spontaneous activity over a large range of spatial, temporal and spectral scales. Slow electrophysiological activity as it is derived from the envelope or power of a limited range of frequencies, also called band limited power (BLP), is of great interest for three reasons. First, changes occur over similar time scales as the BOLD signal. Secondly, it is related to large scale spontaneous oscillations observed between any pair of distant brain regions. Finally, they reflect intrinsic coupling modes that are closely related to structural connectivity and appear relatively constant across brain states (Engel et al., 2013; Woolrich et al., 2013).

The main reason for mapping the sensors to source space, in combination with an atlas based analysis approach, is that it provides a general framework that allows for an anatomical interpretation of the EEG data as well as a direct comparison with other networks derived from fMRI and Diffusion Weighted Imaging (DWI). This is important to allow the extension of our methodology to pathological and atypical brains (Bellec et al., 2010). For example, in epilepsy, localizing accurately and specifically the epileptogenetic zones where seizures initiate is of tremendous importance for the surgical outcome. Current research shows that agreement between EEG and fMRI analysis in detecting the epileptogenetic zone correlates with good surgical outcome (Thornton et al., 2010). Our framework could be extended to shed light on how to interpret observations when there is no multi-modal agreement. For example, examining whether and how the relationship between fMRI and EEG brain networks differ in different brain states and pathological conditions is of particular interest in current clinical neuroscience studies.

### 4.1. Methodological considerations

Sensor level connectivity analysis is biased by the effects of volume conduction/field spread, since there are multiple sensors recording the signal from the same sources. This severely affects the estimation of connectivity and impedes interpretation of the results (Hillebrand et al., 2012). We have used a state of the art approach to estimate the sources from EEG recordings based on beamforming (Brookes et al., 2012). Although, the effect of field spread is not completely abolished, this approach provides a reasonable solution and it is resilient to artifacts in EEG acquired during fMRI such as those due to switched magnetic fields gradients. Another option is to analyse the imaginary part of the coherence, which is robust to volume conductance (Engel et al., 2013). However, functional connectivity based on phase measurements have different interpretations than envelope based connectivity (Engel et al., 2013). It is more variable across brain states and less bound to structural connectivity. The framework provided here can be extended to study both the power envelope and the phase of the EEG signal that could provide valuable insights regarding the connectivity information across modalities.

Our analysis assumes that functional connectivity can be adequately described as a stationary process. Most current connectivity studies assume stationarity to avoid the high complexity involved in modeling the dynamic signal information, which limits the ability to process connectomes with more than 10–20 regions (Smith, 2012). Nevertheless, the extension of our framework, using for example sliding-window correlations, to examine the dynamic complexity of the underlying signals is of particular interest (Brookes et al., 2014). Here, we examine brain connectivity based on the precision matrix, which is the inverse of the covariance matrix and it reflects partial correlation. This is important to disentangle the influence of other regions on each pair-wise connection (Smith et al., 2013) and to allow direct comparison between connectivity variables (Deligianni et al., 2011b, 2013). Partial correlation not only is a reasonable approximation of direct connectivity among brain regions but compared to the usual correlation coefficient it is also more resilient to common underlying noise sources.

The inversion of the covariance matrix requires a well-conditioned SPD matrix. This problem is also known as covariance selection, and in the context of brain connectivity it is challenging due to the problem's intrinsically high dimensional space, and to inter-subject variability (Varoquaux et al., 2010a). In fact, the empirical covariance matrix results in inaccurate estimation of the precision matrix from its inverse due to numerical instabilities and poor estimation of its eigen structure. Here, we use a shrinkage estimator (Krämer et al., 2009) based on the Ledoit and Wolf theorem (Ledoit and Wolf, 2004). This regularizes the estimate of the precision matrix by adding a diagonal matrix to the sample covariance before computing its inverse.

Other approaches to regularizing the inverse covariance matrix based on shrinkage have been recently proposed (Friedman et al., 2008) and they have been suggested in estimating connectivity from fMRI time series (Varoquaux et al., 2010a; Smith et al., 2013). These approaches shrink the estimated values of the precision matrix, so that very small values that are potentially noisy are forced to zero and the rest are better estimated. However, a major challenge is how to determine the shrinkage parameter (Hinne et al., 2014). This is particularly important when we compare connectivity across bands and modalities. One approach is to use cross-validation to choose the shrinkage parameter that best generalizes the estimated covariance within subjects (Pedregosa et al., 2011). This results in connectivity matrices with considerably different sparsity across bands and thus interpretation of the results is not straightforward. Other approaches hypothesize a structure based on prior information provided either from structural data (Deligianni et al., 2013; Hinne et al., 2014) or using population priors (Varoquaux et al., 2010a) and they may introduce strong biases. Furthermore, their extension in populations with neurological diseases is not obvious.

It is important to note that the canonical correlation variables **X** and **Y** that represent EEG and fMRI connectivity, respectively, are not in the form of SPD matrices. They are produced by the concatenation of the vectorized upper triangular matrix of each precision matrix across subjects. The sCCA operates on these connectivity variables based on the lasso *L*_1_ penalty, which results in sparse vectors *u* and *v*. Although there is no explicit constraint to ensure that the prediction will be an SPD matrix, we do not encounter this problem when we predict fMRI from EEG connectomes. On the other hand, when we predict EEG from fMRI connectomes, non-SPD predictions appear on average three times for each cross-validation. This is worse with other approaches of estimating the precision matrix such as the graphical lasso (Friedman et al., 2008; Pedregosa et al., 2011). In this case, most of the predictions are not SPD and therefore we cannot proceed further and estimate the overall prediction performance reliably.

We used gray matter regions derived from standard atlas-based parcellation, which is a common approach (Hillebrand et al., 2012). The main advantage of this whole-brain parcellation is that it is well-defined in subject space and produces corresponding regions across subjects and modalities. It is well known that atlas-based segmentations have poor functional specialization and regions' sizes differ considerably from a few tens of voxels to thousands. This would produce differences in signal to noise ratio of the estimated time-series. Another approach is to use regions drawn from functional studies. Although, these regions are more functionally specialized, there is no universal agreement on how to produce a whole-brain representation and how to propagate it into subject space. We expect that more functionally specialized regions would improve the ability of the proposed approach to select relevant connections and subsequent interpretation of the results (Deligianni et al., 2013).

Nevertheless, our analysis shows that strong inter-hemispheric connectivity between homologous regions is present in both EEG and fMRI connectomes. This is indicated by the lines parallel to the diagonal in the partial correlation matrices, Figure 2. These correlations emerge even though in EEG the voxel time-series to be averaged within a region are drawn randomly for each region and subject. Coupling between homologous sensory areas across hemispheres has been also revealed with envelope correlation in previous seed-based studies (Engel et al., 2013). This is also well established in resting-state fMRI analysis independently of how regions are defined (voxel based or function based) (Biswal et al., 2010). Furthermore, evidence shows that inter-hemispheric connectivity has critical significance for behavior, indicating an important interaction between homologous regions rather than an effect of averaging dissimilar signals (Carter et al., 2010).

## Funding

Funding for this study comes from EPSRC (EP/J016292/1) and is supported by Great Ormond Street Hospital Biomedical Research Center. Dr Maria Centeno is funded by Action Medical Research grant SP4646.

### Conflict of interest statement

The authors declare that the research was conducted in the absence of any commercial or financial relationships that could be construed as a potential conflict of interest.

## Supplementary material

Table S1 **Freesurfer subcortical and cortical regions used in this work to define brain connectomes**.

Figure S1 **Average functional connectivity matrices across subjects in fMRI and EEG δ, θ, α, β, and γ bands, respectively**. The Hilbert envelope of the EEG signal was averaged within each epoch (AWE), which is equal to the fMRI-TR. Therefore, both fMRI and EEG time series have the same number of time samples. **(A–F)** Depicts the partial correlation within cortical regions (68 × 68), whereas **(G–L)** demonstrates the partial correlation within subcortical regions (82 × 82). The regions are given in Table S1 and they are ordered similarly to their matrix representation. For the cortical regions, the top left matrix quadrant represents connections within left hemisphere (lh), the bottom right represent connections within the right hemisphere (rh), and the remaining quadrants represent inter-hemispheric connections. At **(G–L)**, the subcortical regions have been added at the top left corner of the connectivity matrices. This figure is similar to Figure 2 with a noticeable decrease of the partial correlation across all EEG bands. This may be due to less time samples that would result in under-estimation of true connectivity due to the regularization.

Figure S2 **The connections with the 15% highest absolute value in Figure S1 (AWE) are shown as 3D graphs in standard space**. Connections are represented as cylinders with salmon color when they are positive and slate-gray when they are negative. Brain regions are represented with spheres. Their centers and radii represent the centers of mass of each underlying region and its volume, respectively. The color-coding corresponds to different parts of the brain. **(A–F)** Shows partial correlation networks within cortical regions, whereas **(G–L)** shows partial correlation networks within cortical and subcortical regions.

Figure S3 **Results derived from randomized Lasso for the AWE case**. These reflect the 2% connections with the highest probability to be selected overall sCCA repetitions. **(A–F)** Shows results with the precision matrices derived based on cortical regions only, whereas **(G–L)** shows the results obtained with precision matrices that include both cortical and subcortical regions.
