# Supplementary material for: Relating resting-state fMRI and EEG whole-brain connectomes across frequency bands
Source: Front Neurosci. 2014 Aug 28;8:258. doi: 10.3389/fnins.2014.00258 (PMC4148011; doi:10.3389/fnins.2014.00258)
Supplement: Table S1 — Freesurfer subcortical and cortical regions used in this work to define brain connectomes. [file DataSheet1.ZIP › Table S1.PDF]

ROIs Labels	Lobes	ROIs Labels	ROIs Labels	Lobes
lh-thalamus	Subcortical	lh-bankssts	rh-bankssts	Temporal
lh-caudate	Subcortical	lh-caudalanteriorcingulate	rh-caudalanteriorcingulate	Limbic
lh-putamen	Subcortical	lh-caudalmiddlefrontal	rh-caudalmiddlefrontal	Frontal
lh-pallidum	Subcortical	lh-cuneus	rh-cuneus	Occipital
lh-hippocampus	Subcortical	lh-entorhinal	rh-entorhinal	Temporal
lh-amygdala	Subcortical	lh-fusiform	rh-fusiform	Temporal
lh-accumbens	Subcortical	lh-inferiorparietal	rh-inferiorparietal	Parietal
rh-thalamus	Subcortical	lh-inferiortemporal	rh-inferiortemporal	Temporal
rh-caudate	Subcortical	lh-isthmuscingulate	rh-isthmuscingulate	Limbic
rh-putamen	Subcortical	lh-lateraloccipital	rh-lateraloccipital	Occipital
rh-pallidum	Subcortical	lh-lateralorbitofrontal	rh-lateralorbitofrontal	Frontal
rh-hippocampus	Subcortical	lh-lingual	rh-lingual	Occipital
rh-amygdala	Subcortical	lh-medialorbitofrontal	rh-medialorbitofrontal	Frontal
rh-accumbens	Subcortical	lh-middletemporal	rh-middletemporal	Temporal
		lh-parahippocampal	rh-parahippocampal	Limbic
		lh-paracentral	rh-paracentral	Frontal
		lh-parsopercularis	lh-parsopercularis	Frontal
		lh-parsorbitalis	rh-parsorbitalis	Frontal
		lh-parstriangularis	rh-parstriangularis	Frontal
		lh-pericalcarine	rh-pericalcarine	Occipital
		lh-postcentral	rh-postcentral	Parietal
		lh-posteriorcingulate	rh-posteriorcingulate	Limbic
		lh-precentral	rh-precentral	Frontal
		lh-precuneus	rh-precuneus	Parietal
		lh-rostralanteriorcingulate	rh-rostralanteriorcingulate	Limbic
		lh-rostralmiddlefrontal	rh-rostralmiddlefrontal	Frontal
		lh-superiorfrontal	rh-superiorfrontal	Frontal
		lh-superiorparietal	rh-superiorparietal	Parietal
		lh-superiortemporal	rh-superiortemporal	Temporal
		lh-supramarginal	rh-supramarginal	Parietal
		lh-frontalpole	rh-frontalpole	Frontal
		lh-temporalpole	rh-temporalpole	Temporal
		lh-transversetemporal	rh-transversetemporal	Temporal
		lh-insula	rh-insula	Insular
