# Supplementary figures and images for: Relating resting-state fMRI and EEG whole-brain connectomes across frequency bands
Source: Front Neurosci. 2014 Aug 28;8:258. doi: 10.3389/fnins.2014.00258 (PMC4148011; doi:10.3389/fnins.2014.00258)

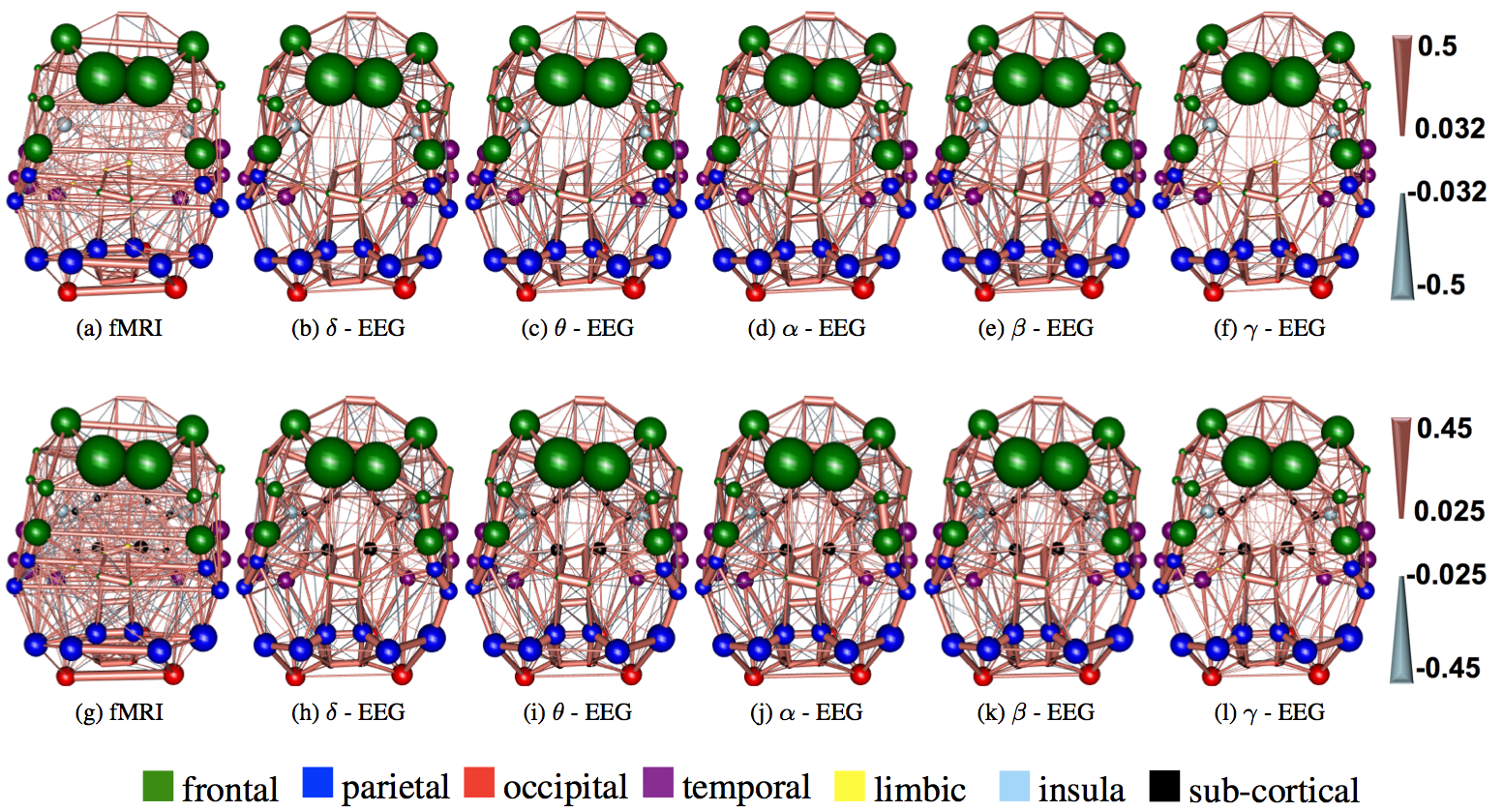

Supplement: Table S1 — Freesurfer subcortical and cortical regions used in this work to define brain connectomes. [file DataSheet1.ZIP › Figure S2.TIFF]

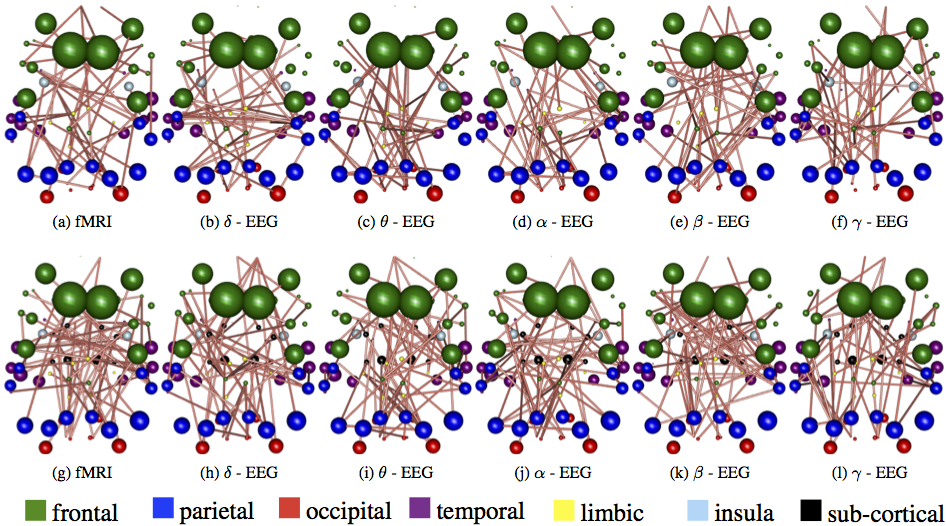

Supplement: Table S1 — Freesurfer subcortical and cortical regions used in this work to define brain connectomes. [file DataSheet1.ZIP › Figure S3.TIFF]

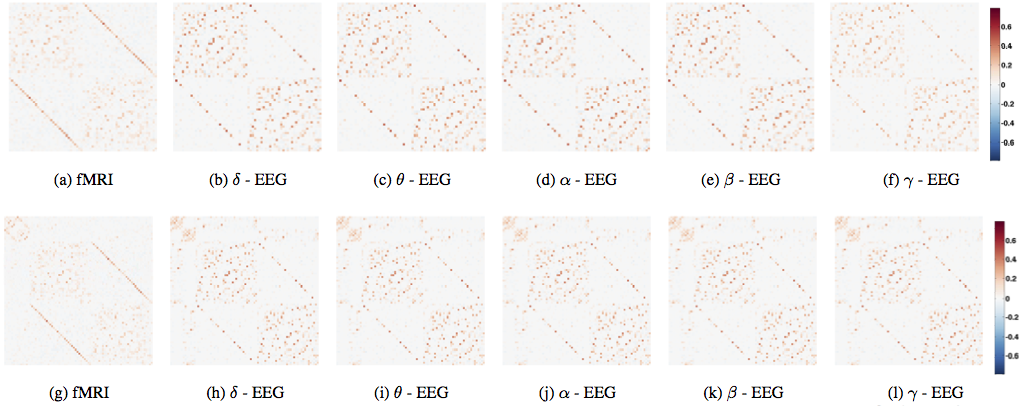

Supplement: Table S1 — Freesurfer subcortical and cortical regions used in this work to define brain connectomes. [file DataSheet1.ZIP › Figure S1.TIFF]
